# Leukotoxic Activity of *Aggregatibacter actinomycetemcomitans* and Periodontal Attachment Loss

**DOI:** 10.1371/journal.pone.0104095

**Published:** 2014-08-05

**Authors:** Carola Höglund Åberg, Dorte Haubek, Francis Kwamin, Anders Johansson, Rolf Claesson

**Affiliations:** 1 Division of Molecular Periodontology, Department of Odontology, Faculty of Medicine, Umeå University, Umeå, Sweden; 2 Section for Pediatric Dentistry, Department of Dentistry, Health, Aarhus University, Aarhus, Denmark; 3 Dental School University of Ghana, Accra, Ghana; 4 Oral Microbiology, Department of Odontology, Faculty of Medicine, Umeå University, Umeå, Sweden; University of Toronto, Canada

## Abstract

*Aggregatibacter actinomycetemcomitans* is a Gram-negative periodontitis-associated bacterium that expresses a toxin that selectively affects leukocytes. This leukotoxin is encoded by an operon belonging to the core genome of this bacterial species. Variations in the expression of the leukotoxin have been reported, and a well-characterized specific clonal type (JP2) of this bacterium with enhanced leukotoxin expression has been isolated. In particular, the presence of the JP2 genotype significantly increases the risk for the progression of periodontal attachment loss (AL). Based on these findings we hypothesized that variations in the leukotoxicity are linked to disease progression in infected individuals. In the present study, the leukotoxicity of 239 clinical isolates of *A. actinomycetemcomitans* was analysed with different bioassays, and the genetic peculiarities of the isolates were related to their leukotoxicity based on examination with molecular techniques. The periodontal status of the individuals sampled for the presence of *A. actinomycetemcomitans* was examined longitudinally, and the importance of the observed variations in leukotoxicity was evaluated in relation to disease progression. Our data show that high leukotoxicity correlates with an enhanced risk for the progression of AL. The JP2 genotype isolates were all highly leukotoxic, while the isolates with an intact leukotoxin promoter (non-JP2 genotypes) showed substantial variation in leukotoxicity. Genetic characterization of the non-JP2 genotype isolates indicated the presence of highly leukotoxic genotypes of serotype b with similarities to the JP2 genotype. Based on these results, we conclude that *A. actinomycetemcomitans* harbours other highly virulent genotypes besides the previously described JP2 genotype. In addition, the results from the present study further highlight the importance of the leukotoxin as a key virulence factor in aggressive forms of periodontitis.

## Introduction

Periodontitis is an inflammatory disease, initiated by a bacterial biofilm on the teeth and characterized by the degradation of the tooth-supporting tissues [1]. The disease usually progresses slowly among adults, whilst in young individuals rapidly progressing forms of the disease are usually seen. The bacterium, *Aggregatibacter actinomycetemcomitans*, is strongly associated with the aggressive forms of the disease [2]. Based on longitudinal studies, the presence of *A. actinomycetemcomitans* is considered a risk marker for the progression of periodontal attachment loss (AL), i.e. degradation of the periodontal tissues around the teeth [3], [4], [5]. While a cytolethal distending toxin (CDT) produced by this bacterial species seems to have limited effect on disease progression, the opposite has been reported for the leukotoxin, another toxin produced by the bacterium [6]. A specific genotype of *A. actinomycetemcomitans*, JP2, with the capacity to produce substantial amounts of the leukotoxin, has been shown to be highly associated with aggressive forms of periodontitis [5], [7].

The leukotoxin exerts its effect on the immune defense cells in different ways [8]. It binds to the lymphocyte function-associated antigen-1 (LFA-1) molecule of haematopoetic cells and induces an activation and secretion of lysosomal proteases from neutrophils and IL-1β from macrophages [9], [10], [11]. This leukotoxin-induced cellular activation subsequently causes death of the target cells. It has also been shown that leukotoxin induces β-haemolysis in erythrocytes, although these cells lack the LFA-1 molecule on the cell surface [12], [13].

The leukotoxin operon consists of the *ltxC, ltxA, ltxB*, and *ltxD* genes and a promoter region [14], [15]. The *ltxA* codes for the protein, while the *ltxC* encodes components responsible for the posttranslational acylation. The two other genes, the *ltxB* and the *ltxD*, are involved in the transport of the leukotoxin to the bacterial surface [14], [15], [16].

The leukotoxicity varies among different strains of *A. actinomycetemcomitans.* The presence of specific sequences within the promoter region and the binding of regulation proteins to these sequences are thought to control the expression of the leukotoxin [17], [18], [19]. Environmental factors, such as levels of oxygen or fermentable sugars, have also been shown to regulate the leukotoxicity [20], [21], [22]. High levels of leukotoxin production have been detected in *A. actinomycetemcomitans* strains with a 530-base pair (bp) deletion within the leukotoxin promoter region [23], [24]. Strains with such truncated promoter regions are described as JP2-like or JP2 clonal strains [24].

The JP2 genotype is thought to have originated in North Africa, spread to West Africa and then spread among individuals of African descent living in South America, North America and Europe [7], [25], [26], [27]. Reports on the carriage of the JP2 genotype of *A. actinomycetemcomitans* in Caucasians are scarce, but a few exist [28], [29].

Individuals carrying the JP2 genotype of *A. actinomycetemcomitans* have a significantly higher risk for progression of AL than carriers of the non-JP2 genotypes [5]. It has been suggested that this enhanced risk for disease progression is due to the higher leukotoxicity of the JP2 genotype compared with the non-JP2 genotypes [24], [30]. In other words, the prevailing opinion is that the JP2 genotype is highly leukotoxic, while non-JP2 genotypes, strains with a complete leukotoxin promoter, are minimally leukotoxic. However, since the majority of individuals developing rapid bone loss at a young age are colonized with non-JP2 genotypes, it seems that the usage of methods which only distinguish between JP2 and non-JP2 genotypes provides too little information on the actual virulence capacity of the various clones of the bacterium.

Therefore, in the present study we focus on the *de facto* variations in leukotoxicity in different genotypes of *A. actinomycetemcomitans*. We hypothesize that the leukotoxic activity of this bacterial species is of importance for the progression of periodontitis of the infected individuals, and that the leukotoxicity can be used as a virulence marker.

## Results

### Leukotoxicity of different *A. actinomycetemcomitans* isolates

For studying the leukotoxicity of clinical isolates of *A. actinomycetemcomitans*, we used two established methods, one based on cell lysis and the other based on cell death. We also tested if haemolysis that is induced when *A. actinomycetemcomitans* is grown on blood agar can be used as a leukotoxin assay [12].

**Figure 1:**
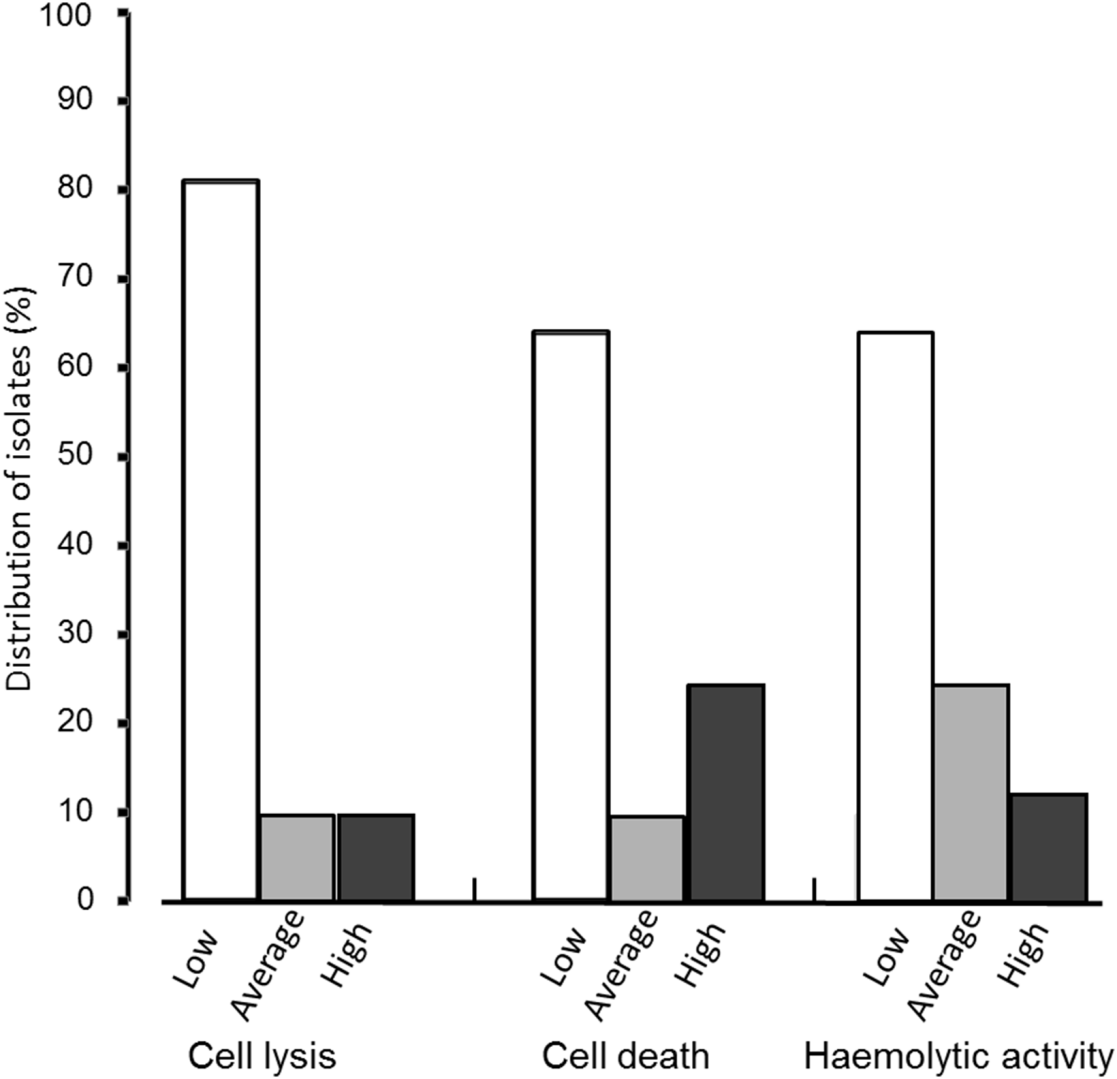
Distribution of *A. actinomycetemcomitans* isolates into three categories according to their leukotoxicity (n = 239). Cell lysis (LDH) and cell death (NRU) assay: 1 = minimal leukotoxicity (0–30%), 2 = average leukotoxicity (31–60%), 3 = high leukotoxicity (≥61%). Haemolysis-based assay: 1 = minimal leukotoxicity (no or small zone), 2 = average leukotoxicity (average zone), 3 = high leukotoxicity (large zone).

**Figure 2:**
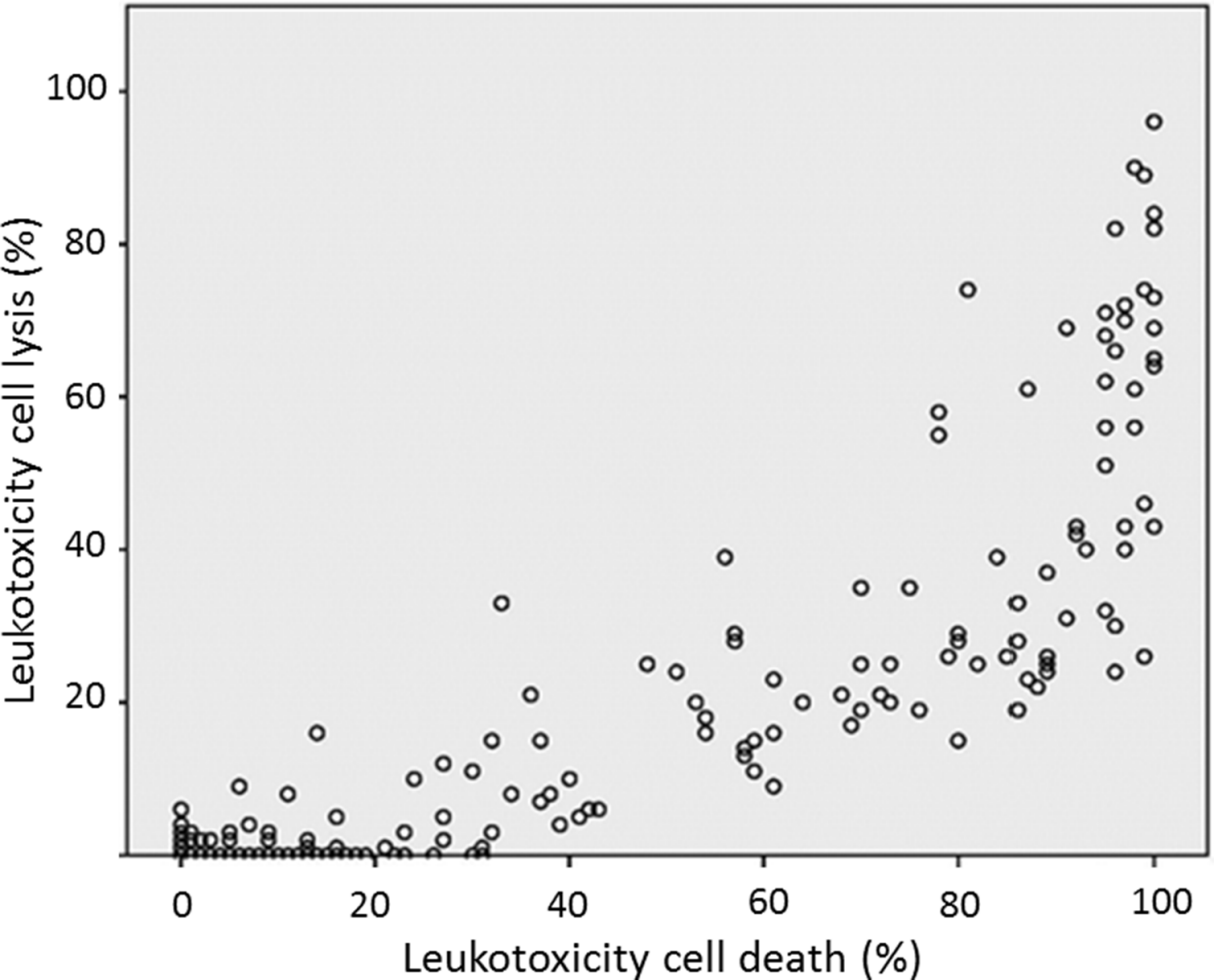
Correlation between the cell lysis (LDH) method and the cell death (NRU) method according to leukotoxicity in isolates (n = 239).

**Figure 3:**
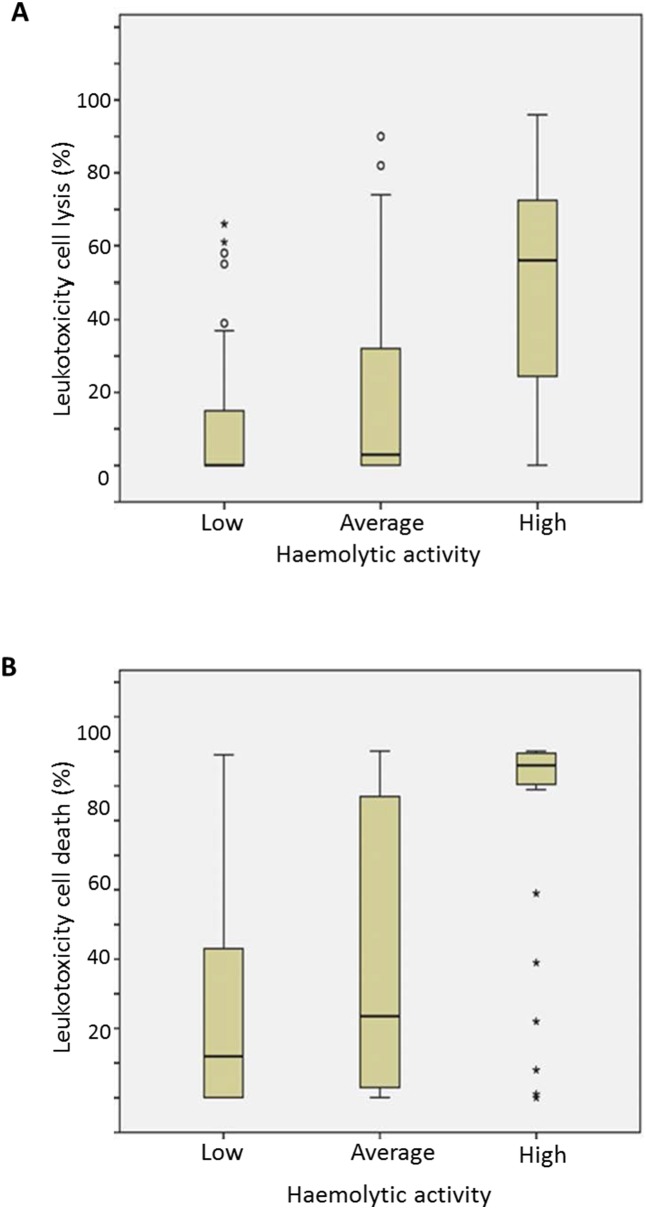
Distribution of *A*. *actinomycetemcomitans* isolates into three categories (minimal, average, high) according to leukotoxicity (n = 239). **A.** Correlation between the cell lysis (LDH) method and the haemolysis method. **B.** Correlation between the cell death (NRU) method and the haemolysis method.

We found that the majority of the isolates (n = 239) showed low leukotoxicity (Fig. 1). Using the cell lysis method (LDH release), a smaller proportion of the isolates were placed in the highly leukotoxic category than when the cell death method (NRU) was used (Fig. 1). This discrepancy between the two methods is further illustrated in a scatter plot diagram (Fig. 2). This figure indicates that the majority of the isolates that show average leukotoxicity (31–60%) with the cell lysis method (LDH), show a high level of leukotoxicity (>61%) with the cell death (NRU) method. This figure shows that the majority of the isolates that show average leukotoxicity (31–60%) with the cell lysis method (LDH) roughly correspond to the isolates that show high leukotoxicity (≥61%) with the cell death (NRU) method. The results were correlated from the three different tests (leukotoxin assay based on haemolytic activity, cell lysis assay (LDH), and cell death (NRU) assay) (Fig. 2 and Fig. 3A, B).

**Figure 4:**
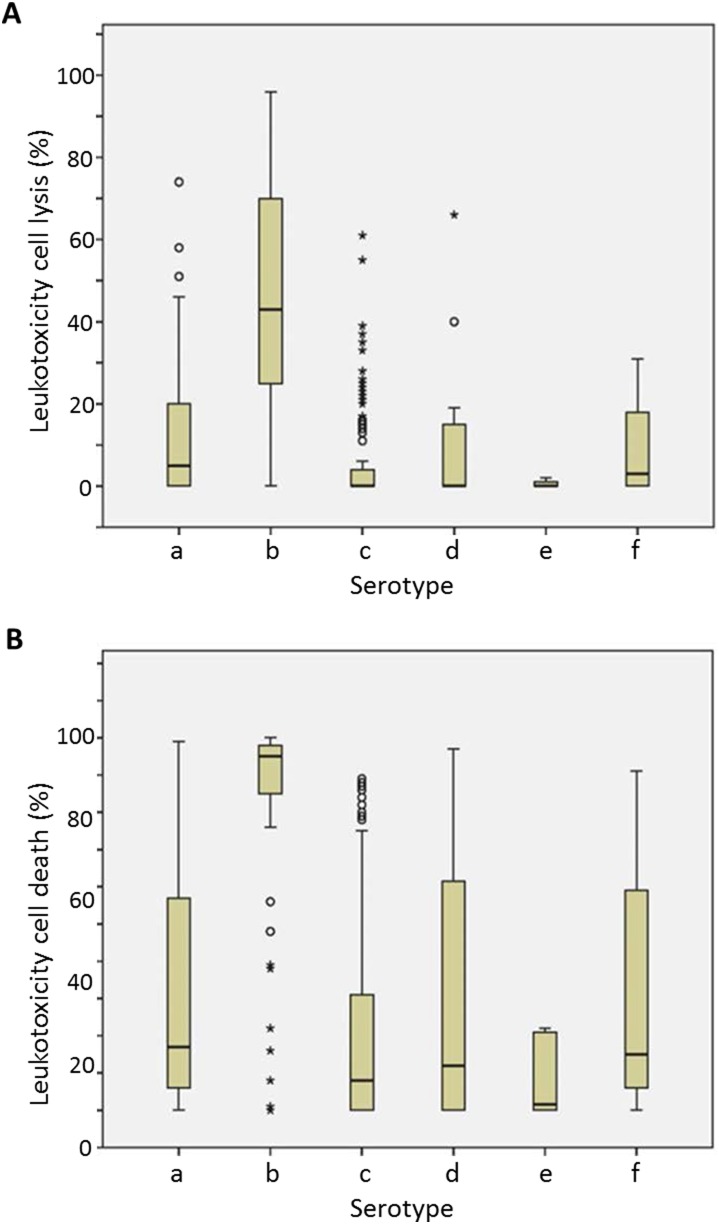
Leukotoxicity of various serotypes of *A. actinomycetemcomitans* (n = 239) determined by **A.** The cell lysis (LDH) method. **B.** The cell death (NRU) method.

When focusing on the leukotoxicity of the serotypes a to f, we found diversity both between and within the different serotypes. Most striking was that serotype b isolates were represented by both highly leukotoxic and low leukotoxic isolates. These results were determined by the cell lysis (LDH) methd and the cell death (NRU) method (Fig. 4A, B). The results of the haemolysis-based leukotoxicity assay were consistent with this (Table 1).

**Table 1:** Haemolytic activity of various serotypes (n = 239) of *A. actinomycetem-comitans* determined by the haemolysis method.

	minimal	average	high	total
*serotypes*	*n (%)*	*n (%)*	*n (%)*	*n*
a	43 (75.4)	14 (24.6)		57
b	1 (2.2)	18 (39.1)	27 (58.7)	46
c	83 (83.0)	17 (17.0)		100
d	10 (83.3)	2 (16.7)		12
e	5 (83.3)	1 (16.7)		6
f	10 (55.5)	7 (38.9)	1 (5.6)	18

**Figure 5:**
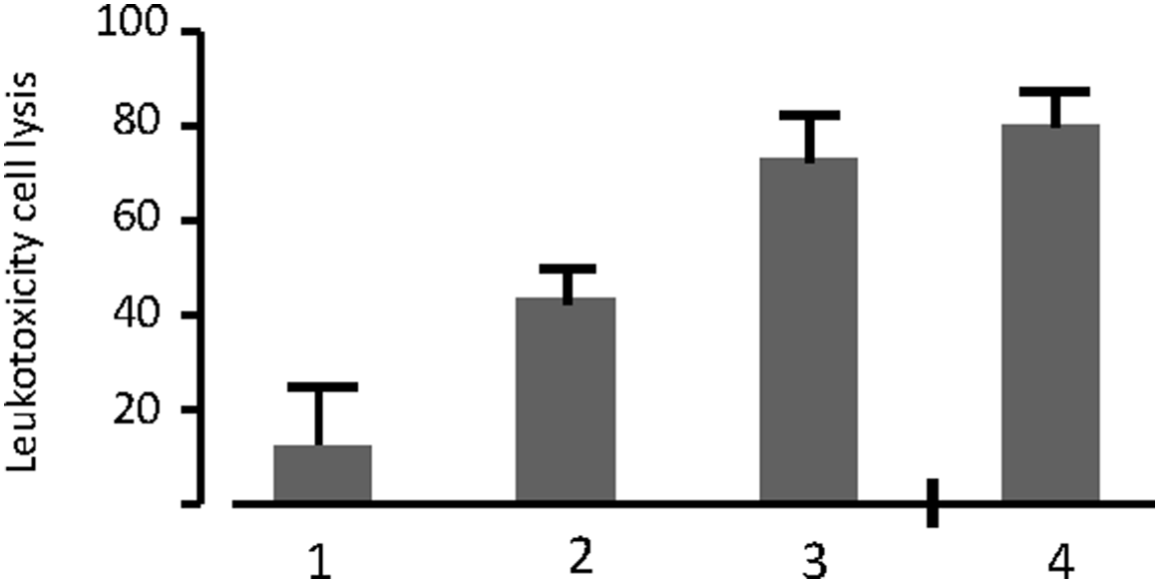
Distribution of serotype b isolates (n = 51) in various leukotoxicity categories based on data obtained by the cell lysis (LDH) method. **1.** Minimal leukotoxicity, serotype b, non-JP2 genotype isolates (n = 15), **2.** Average leukotoxicity, serotype b, non-JP2 genotype isolates (n = 11) **3.** High leukotoxicity, serotype b, non-JP2 genotype isolates (n = 15), **4.** JP2 genotype isolates (n = 10). Mean LDH-release ± SD.

As determined by the cell lysis (LDH) method, fifteen (36.6%) out of the 41 non-JP2 genotype isolates, serotype b were classified as highly leukotoxic and expressed leukotoxicity similar to that of the 10 JP2 genotype isolates examined (Fig. 5).

### Sequencing of the leukotoxin promoter

The differences in the leukotoxicity of *A. actinomycetemcomitans* strains have been suggested to be due to variations in the leukotoxin promoter sequence [17]. When we sequenced the promoter region of the 41 non-JP2 genotype serotype b isolates, only three sequence types (ST) were identified: ST 1 (n = 39), ST 2 (n = 1), and ST 3 (n = 1). The polymorphic sites which define the sequence types are shown in Table 2.

**Table 2:** Polymorphic sites in the leukotoxin promoter region of serotype b isolates (n = 41).

ST	n	-1	-79	-219	-220	-221	-436	-646	-1109
1	39	T	A	A	T	A	C	C	T
2	1	T	A	A	T	A	C	T	T
3	1	T	A	*	*	*	T	C	T

The leukotoxin promoter sequence of the isolates was compared with the corresponding sequence of the reference strain Y4 (accession number HAU51861). Y4 sequence was identical to ST 3.

ST (sequence types). Position -1 corresponds to the first nucleotide upstream of the start codon for *lkt.* Position – 1109 corresponds to the first nucleotide after the terminal codon of *glyA.*

*Deletion of a single nucleotide at this position.

Isolates of ST2 and ST3 were found to have average and minimal leukotoxicity, respectively. These findings indicate that the differences in the leukotoxicity between these Ghanaian isolates are not due to the genetic organization of the promoter of the leukotoxin operon.

The sequence of the undeleted part of the promoter of the five JP2 strains isolated at baseline was identical to the corresponding part of the promoter of ST 1 and ST 3-isolates and of Y4, i.e., all differences within the promoter sequence of 40 (ST 1 and ST 3) of the Ghanaian isolates were located in the part of the promoter that is deleted in the JP2 genotype of *A. actinomycetemcomitans*.

### Sequencing of the *hbpA-2* gene

Among the identified nucleotide mutations within *hbpA*-2, one has been suggested to distinguish JP2 isolates of North African and West African origin [25]. When we sequenced this particular gene in the 10 JP2 genotype isolates included in the present study, we found the previously reported point G to A SNP (position 525285 in the HK 1651 genome) which indicates a West-African origin of the Ghanaian JP2 genotype isolates.

### Characterization of non-JP2 and JP2 genotypes by Arbitrarily primed (AP) PCR

**Figure 6:**
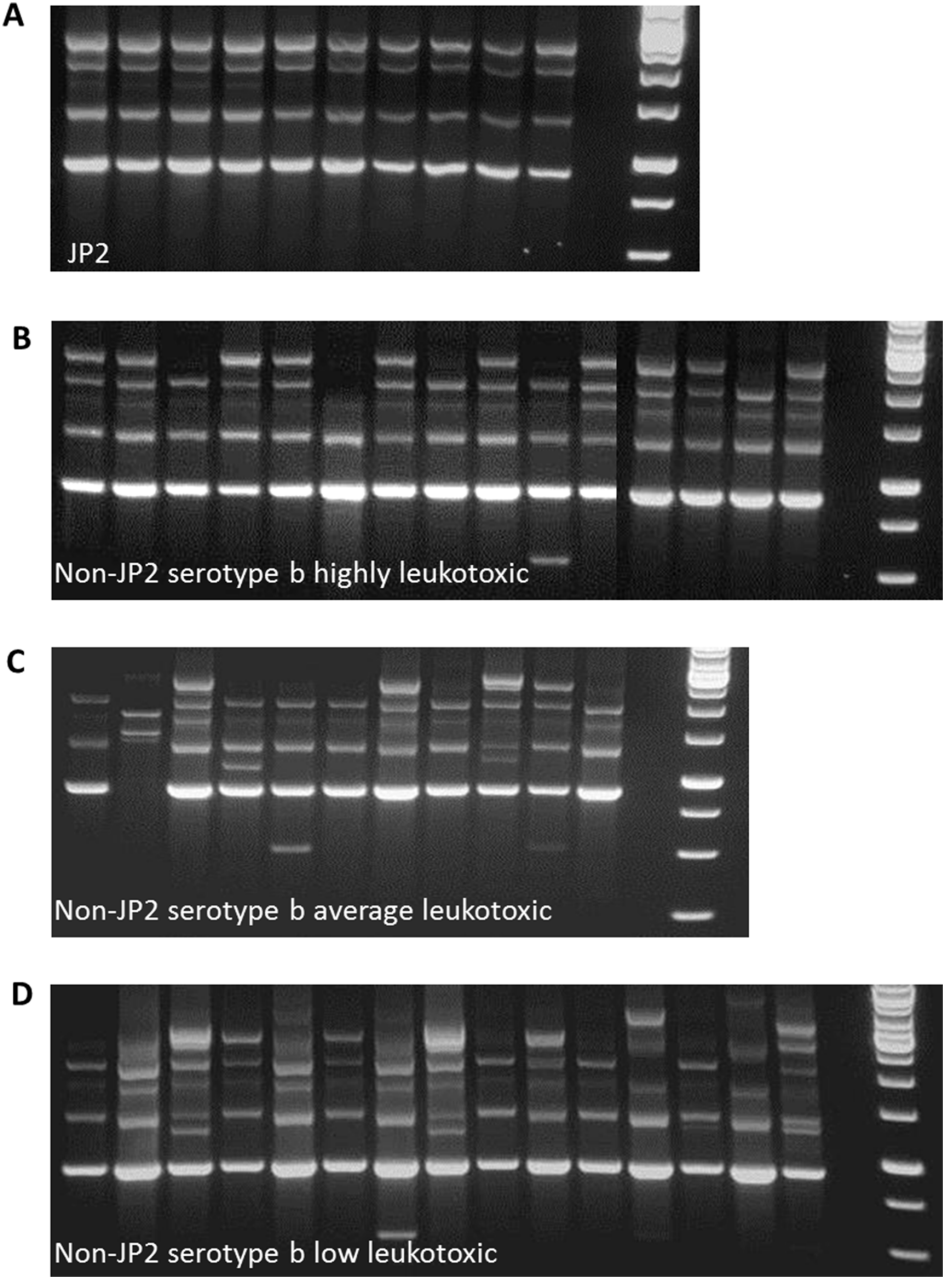
AP PCR banding pattern of **A.** JP2 genotype isolates (n = 10), **B.** High leukotoxicity non-JP2 genotype isolates of serotype b (n = 15), **C.** Average leukotoxicity non-JP2 genotype isolates of serotype b (n = 11), **D.** Minimal leukotoxicity non-JP2 genotype isolates of serotype b (n = 15). The classification of the non-JP2 genotype serotype-b isolates is based on results from the cell lysis (LDH) method.

When we used the AP PCR technique to study our *A. actinomycetemcomitans* isolates of serotype b, it revealed >10 different banding patterns (Fig. 6A–D). The patterns produced by the 10 JP2 genotype isolates were identical (Fig. 6A). Interestingly, most of the non-JP2 genotype isolates distributed from the highly leukotoxic cell lysis (LDH) category exhibited a pattern identical to that of the JP2 genotype isolates (Fig. 6, Table 3). The banding pattern was more diverse among the isolates of the average and minimal leukotoxicity cell lysis (LDH) groups (Fig. 6, Table 3).

**Table 3:** AP PCR types with serotype b isolates (n = 51) divided into leukotoxicity categories based on results of the cell lysis (LDH) method.

	AP PCR types	
Genotypes of A.a*	JP2/JP2-like, n (%)	Other, n (%)
JP2	10 (100)	0 (0)
non-JP2 high leukotoxicity	10 (67)	5 (33)
non-JP2 average leukotoxicity	2 (18)	9 (82)
non-JP2 minimal leukotoxicity	2 (13)	13 (87)

*A.a; A. actinomycetemcomitans.

*(%): proportion of isolates within each leukotoxicity group.*

### Leukotoxicity and increased risk for progression of AL

The leukotoxicity of *A. actinomycetemcomitans* isolates obtained from a subgroup of the study population, consisting of 146 adolescents living in Ghana, was studied (Table 4). We also evaluated the risk for a progression of AL≥3 mm in 146 of these individuals in relation to the carriage of the different genotypes of *A. actinomycetemcomitans* with varying leukotoxicity. The isolates examined were grouped into categories of minimal, average and high leukotoxicity when the cell lysis (LDH) and the cell death (NRU) methods were used. In the analysis of the risk (OR) for the progression of AL at the individual level (Table 4), the results from the cell lysis (LDH)- and the cell death (NRU)-methods were combined into two categories. In order to represent individuals that were carriers of strains with high leukotoxicity, the average category for the cell lysis (LDH) method was combined with the high leukotoxicity category, and the average category was combined with the minimal leukotoxicity category when the more sensitive cell death (NRU) method was used (Table 4).

**Table 4:** Odds ratio (OR) for the progression of attachment loss (AL) according to leukotoxicity.

Variable	OR	95% CI	*p*-values	Individuals,total number(n)	Individuals, withprogression ofAL≥3 mm, n (%)
*Cell lysis*					
minimal	4.1	2.3–7.3	<0.001	110	41 (37.3)
Average + high	10.9	4.9–24.1	<0.001	36	22 (61.1)
*Cell death*					
Minimal + average	4.3	2.4–7.7	<0.001	97	37 (38.1)
high	7.8	3.9–15.8	<0.001	49	26 (53.1)
*Haemolysi*s					
minimal	4.8	2.6–8.8	<0.001	90	37 (41.1)
average	3.9	1.8–8.5	<0.001	39	14 (35.9)
high	16.6	5.4–51.3	<0.001	17	12 (70.9)
*A.a* * *negative*	1.0	reference		190	24 (12.6)

Cell lysis (minimal = 0–30%, *average + high* = ≥31% release of cytosolic LDH), Cell death (*minimal + average* = 0–60%, *high* = ≥61% reduction of viable cells), Haemolysis (1 = minimal, none or small zone, 2 = *average*, average size zone, 3 = *high*, large size zone). Subgroup of individuals (n = 146) positive for *A. actinomycetemcomitans*. The reference group consisted of individuals (n = 190) who were negative for *A. actinomycetemcomitans.* OR, odds ratio; confidence interval; AL, attachment loss. Significance (*p*<0.05).

*A.a; A. actinomycetemcomitans.

A significantly increased risk of progression of AL≥3 mm was found among individuals who carried *A. actinomycetemcomitans* in relation to the reference group (the *A. actinomycetemcomitans*-negative individuals) (Table 4). Those individuals with a notably increased risk (OR>10) of developing progression of AL were colonized with average to highly leukotoxic *A. actinomycetemcomitans* (Table 4). Consequently, highly leukotoxic *A. actinomycetemcomitans* correlated most strongly with the progression of AL. Corresponding results were obtained when the semi-quantitative haemolysis-based leukotoxin assay was used, i.e. the carriage of highly haemolytic genotypes (category 3) was strongly associated with the progression of AL≥3 mm (Table 4).

After exclusion of the carriers harbouring the JP2 genotype, the remaining carriers of the highly leukotoxic *A. actinomycetemcomitans* were still at a significantly enhanced risk for disease progression. An exclusion of the JP2 genotype-positive individuals in the “minimal LDH” category resulted in an OR of 3.9 (95% CI [2.1–7.0], *p*<0.001), and in the “average–high (LDH)” category the OR was 6.9 (95% CI [2.9–16.2], *p*<0.001). In the “minimal–average” category according to the cell death (NRU) method, the exclusion resulted in an OR of 4.1 (95% CI [2.2–7.6], *p*<0.001), and in the “high (NRU)” category the OR was 5.1 (95%CI [2.4–10.9], *p*<0.001). Exclusion of the carriers with the JP2 genotype in the “minimal haemolysis” category, resulted in an OR of 4.2 (95% CI [2.3–7.8], *p*<0.001), in the “average haemolysis” category the OR was 3.5 (95% CI [1.5–8.0], *p* = 0.004), and in the “high haemolysis” category the OR was 11.1 (95% CI [3.3–36.6], *p*<0.001). For the cell lysis (LDH) method, we consider isolates that cause ≥31% cell lysis (LDH) as having an enhanced leukotoxic activity, and these were all classified as highly leukotoxic when examined by the cell death (NRU) method (Fig. 2).

In the present study population (n = 146), 32 out of 41 carriers of the non-JP2 genotypes of *A. actinomycetemcomitans* of serotype b were clinically examined at the follow-up two years later. Among 20 of these examined individuals having a progression of AL>3 mm, 14 (70%) carried *A. actinomycetemcomitans* with a leukotoxicity ≥31%. In contrast, among 12 of the examined individuals with a progression of AL<3 mm, only 5 (40%) were colonized with *A. actinomycetemcomitans* belonging to the category with high leukotoxicity.

## Discussion

In the present study we have shown that individuals harbouring highly leukotoxic *A. actinomycetemcomitans*, independently of carrying a complete or truncated (JP2-genotype) leukotoxin promoter, had a significantly increased risk of progression of AL. We observed that the non-JP2 genotype isolates with high leukotoxicity comprised a subgroup of serotype b, and we found that the sequence of the leukotoxin promoter region in the non-JP2 genotype isolates of serotype b was similar, independent of leukotoxicity of the isolates. Taken together, these findings provide new insights into the leukotoxin as a virulence factor. As other genotypes than the JP2 genotype are shown to be highly leukotoxic, the association of *A. actinomycetemcomitans* to disease should be considered in a broader perspective than previously.

Based on our findings, we suggest that the generally accepted concept that *A. actinomycetemcomitans* can be divided into two categories based on leukotoxicity and organization of the leukotoxin promoter region should be further developed. In our point of view, the JP2 genotype of *A. actinomycetemcomitans*, characterized by a leukotoxin promoter region missing a 530-bp fragment, is highly leukotoxic [24]. This is further supported by reports that individuals carrying the JP2 genotype of *A. actinomycetemcomitans* are at increased risk for the development of AL [5], [31].

However, in the present study, we have shown that there is more extensive variation in leukotoxicity among genotypes with a complete leukotoxin promoter than earlier assumed. Our results also show that the progression of AL in infected individuals is associated with the leukotoxicity (minimal, average or high) of *A. actinomycetemcomitans*, as determined *in vitro*. This indicates that analysis of virulence characteristics of *A. actinomycetemcomitans* should not be limited to leukotoxin promoter typing but should also include the determination of leukotoxic activity.

*A. actinomycetemcomitans* of serotype b has been reported to be associated with an increased risk for the development of periodontitis for decades [32], [33]. Higher leukotoxicity within serotype b, as compared with the other serotypes, has been suggested to be one reason [30]. The present study shows that there is a substantial variation in leukotoxicity among serotype-b strains. We identified isolates of serotype b that exert high leukotoxicity as well as some that showed no leukotoxic activity at all. The most striking observation was that the highly leukotoxic group of serotype b comprised both JP2 and non-JP2 genotype isolates. This means that more than one highly leukotoxic genotype can be found within the b serotypes. Based on this novel finding it would be valuable if focus were moved from distinguishing between JP2 and non-JP2 genotypes, to also include determination of the actual leukotoxicity of *A. actinomycetemcomitans,* serotype b.

In order to further study the variation in the leukotoxicity among *A. actinomycetemcomitans* isolates of serotype b, the leukotoxin promoter region of the serotype b isolates was sequenced. However, almost no differences in the organization of the promoter region among the isolates were observed. This indicates that the organization of the promoter region was not responsible for the observed variations in leukotoxicity. However, the AP PCR-based characterization of the isolates revealed different banding patterns. As expected, the JP2 genotype isolates had an identical banding pattern. Interestingly, the majority of the isolates with high leukotoxicity showed a ‘JP2-like’ pattern. The AP PCR banding pattern of the remaining isolates was more diverse. The AP PCR method has previously been used for the characterization of *A. actinomycetemcomitans* strains isolated from healthy and diseased individuals [34], [35], [36]. For the identification of putative virulence genes, the AP PCR technique provides limited information. However, our observations indicate that screening for highly leukotoxic non-JP2 genotypes of *A. actinomycetemcomitans* of serotype b might be successful if extended gene level comparisons between different *A. actinomycetemcomitans* isolates of serotype b are performed.

For the determination of leukotoxicity, we used three different assays. Based on the results from the cell lysis (LDH) and the cell death (NRU) methods, the isolates were categorized into groups with high, average, or minimal leukotoxicity. Although the two methods gave similar results, fewer isolates were identified as highly leukotoxic by the cell lysis (LDH) method than with the cell death (NRU) method. Taken together, we concluded that the cell lysis (LDH) method seemed to identify the “true” highly leukotoxic isolates with greater accuracy than the cell death (NRU) method. However, this latter method identified low leukotoxic producers which otherwise could have been overlooked by the cell lysis (LDH) method.

The JP2 genotype of *A. actinomycetemcomitans* induces β-haemolysis on blood agar plates [12], [37]. As far as we know, this is the first study in which haemolytic activity has been used for the determination of the leukotoxicity of clinical *A. actinomycetmcomitans* isolates. The results were in accordance with those achieved by the cell lysis (LDH) and cell death (NRU) methods. However, further studies of the relevance of the method are required before it can be recommended as a screening method for highly leukotoxic *A. actinomycetemcomitans* genotypes.

In the present study, we provide new information about the diversity of the *de facto* leukotoxicity within the species *A. actinomycetemcomitans*. The importance of these data was substantially improved since we were able to evaluate the progression of AL in relation to the leukotoxicity of *A. actinomycetemcomitans* isolates collected from healthy or diseased individuals. Previous reports showing the virulence mechanisms of the leukotoxin and the enhanced risk of the progression of AL for individuals carrying the highly leukotoxic genotype (JP2) of this bacterium further strengthen the relevance of the present study [5], [7], [8], [31].

In conclusion, our data provide evidence for a substantial variation in the leukotoxicity among the non-JP2 genotype strains of *A. actinomycetemcomitans* that were often previously grouped into the category of minimally leukotoxic strains. These data clearly support leukotoxicity of *A. actinomycetemcomitans* as an important virulence factor and a causative agent implicated in the progression of periodontitis.

## Materials and Methods

### Ethics statement

Ethical clearance for the study was obtained from the Noguchi Memorial Institute for Medical Research, University of Ghana (IRB 000 1276), and from the local Ethical committee of Umeå University, Sweden (Dnr 2010-188-31M). Signed consents were received from the parents or the guardians of the children before they entered the study.

### Study collection

This study is based on a characterization of 239 *A. actinomycetemcomitans* isolates (234 non-JP2 and 5 JP2 genotypes) obtained from 199 Ghanaian adolescents [38]. This collection is a part of a collection previously described [6].

In addition, based on a subpopulation of 146 individuals we evaluated the potential risk for progression of AL for the carriers of the different genotypes of *A. actinomycetemcomitans* [6].

### Determination of leukotoxic activity

Three different methods were used for analyzing the leukotoxicity of the 239 different isolates: 1) Cell lysis, 2) Cell death, and 3) Haemolytic activity.

#### Cell lysis (LDH)

Leukotoxicity analysis with the cell lysis (LDH) assay reveals loss of membrane integrity determined by the activity of lactate dehydrogenase that is released from damaged THP-1 cells (human acute monocytic leukemia cell line, ATCC 16) after 60-min exposure to the leukotoxic extract [8]. Briefly, each of the 239 isolates was re-cultivated on blood agar until conversion to a smooth phenotype was obtained. The isolates were then cultured in polyethylene glucose broth (PYG, peptone 20 g/l, glucose 5 g/l, Baptist yeast powder 10 g/l, NaCl 0.08 g/l cysteine hydrochloride 0.5 g/l, CaCl_2_ 0.008 g/l, MgSO_4_ 0.008 g/l, KH_2_HPO_4_ 0.04 g/l, K_2_HPO_4_ 0.04 g/l, NaHCO_3_ 0.4 g/l, pH 7.1–7.3) for 72 hours at 37°C, and the optical density (OD_600 nm_) was measured. The bacterial suspensions were centrifuged for 5000×g for 20 min at 4°C, and the pellet was re-suspended in 300 mM NaCl in PBS to a final concentration corresponding to OD_600 nm_ = 20. The THP-1 cells were seeded in 96-well plates with 10^5^ cells/well in the presence of RPMI with 10% fetal calf serum and 50 nM PMA. After 24 h incubation at 37°C in 5% CO_2_, the medium was replaced with 100 µl RPMI with 10% FBS and 5% leukotoxic extract. Extracts from each isolate were analyzed in triplicate, and the mixtures were incubated for 60 min at 37°C in 5% CO_2_. The LDH activity in the supernatants was analyzed through the decreased absorbance at 340 nm as a result of NADH oxidation catalyzed by the release of cytosolic LDH. The release of LDH was expressed as % of the maximal release (100%) caused by incubation with 0.1% Triton x-100. The leukotoxicity of the isolates was classified in relation to their capacity to induce LDH-release: minimal leukotoxicity ≤30%, average leukotoxicity ≥31–60% or high leukotoxicity ≥61% release of cytosolic LDH.

#### Cell death (NRU)

The cell viability assay (NRU) quantifies viability in relation to the capacity to accumulate neutral red in the lysosomes of leukotoxin-exposed THP-1 cells. The accumulation of neutral red in the lysosomes requires cells with an intact proton potential in the lysosomal membrane [39]. THP-1 cells and the leukotoxic extracts were processed as described for the cell lysis (LDH) assay. After a 60-min incubation period with the leukotoxic extracts, the culture medium was replaced with 100 µl fresh culture medium containing neutral red. After a 2-h staining period at 37°C in 5% CO_2_, the staining medium was discarded and the cell monolayer was rinsed with 150 µl PBS. The cells were dissolved in 150 µl lysis solution (50% ethanol with 1% acetic acid), and the absorbance from the stain was measured at 550 nm. Cell viability (%) was calculated in relation to the absorbance of a sample incubated without leukotoxic extract. The leukotoxicity of the isolates was classified according to their capacity to induce cell death: minimal leukotoxicity ≤30%, average leukotoxicity ≥31–60% or high leukotoxicity ≥61% reduction of viable cells.

#### Haemolytic activity

The haemolytic activity has been suggested to reflect the effect of leukotoxin on erythrocytes. It has not previously been used for quantification of leukotoxicity. The haemolytic activity of the bacterial isolates was measured according to the following protocol. The bacterial isolates were cultivated on blood agar plates (as above) for 3–5 days. Subsequently, 5 µl OD 2-suspensions in 0.9% NaCl (approximately 4×10^9^ cells/ml) of the isolates were added to blood agar plates (Columbia blood agar base, Acumedia, Neogen, Lansing, Michigan, USA) containing 5% horse blood and 10 µM deferoxamine (Sigma Aldrich; St. Louise, MO, USA.). Suspensions from six different isolates and three reference strains (JP2, D7s and a leukotoxin mutant of the D7s strain ([D7S*ΔltxA*]) were applied to each plate. The plates were incubated 5–7 days at 37°C in 5% CO_2_ before examination. The haemolytic activity was scored as high (zone similar in size to that produced by JP2), average (zone similar to that produced by D7s), or low (minimal zone as produced by D7S*ΔltxA).*

#### Estimated risk (OR) of progression of AL in relation to leukotoxicity

To assess the estimated risk (OR) of the progression of AL within individuals harbouring strains from the three leukotoxicity categories (minimal, average or high), with the LDH and NRU-methods, the definition was as follows: The isolate of *A. actinomycetemcomitans*, with the highest leukotoxicity score was determined to be representative for the individual studied. Therefore, if more than one serotype of *A. actinomycetemcomitans* was detected in an individual, the isolate/serotype with the highest score of leukotoxicity was chosen to be representative for that individual.

### PCR-based characterization

Before the PCR-based analyses were performed, DNA was purified with GenElute Bacterial DNA kit (Sigma-Aldrich, St. Louise, MO, USA). For preparation of the PCR mixtures, PureTaq Ready-To-Go PCR (GE Healthcare; Buckinghamshire, UK) was used. For the sequencing procedure, DNA of amplified genes was sent to Eurofins MWG Operon (Ebersberg, Germany).

#### AP PCR

Arbitrarily-primed (AP) PCR, which can be carried out without previous knowledge of specific DNA sequence, was used for the characterization of the serotype b isolates. For amplification, the random sequence oligonucleotide OPB-3 (AGTCAGCCAC) (Invitrogen, Carlsbad, CA, USA) (0.4 µM) was used. The concentration of MgCl_2_ in the PCR mixture was increased to 2.5 µM. The amplification was carried out as described by Dogan and co-workers [40].

#### Sequencing of the house-keeping gene hbpA-2

By sequencing *hbpA-2*, the origin of the JP2 genotype isolates used in this study could be determined. For amplification the primers TATTTTGACGCAATTGCTGTTC and TAGGGCACTTATCATTTCCATC (0.4 µM each) were used. The amplification was carried out as described by Eriksen and co-workers [41].

#### Sequencing of the leukotoxin promoter region

Sequencing the promoter region of the leukotoxin operon was carried out to determine sequence differences among the serotype b isolates. For amplification primer ltx3 (GCCGACACCAAAGACAAAGTCT’) and primer ltx4 (GCCCATAACCAAGCCACATAC) (0.4 µM each) were used. The amplification was carried out as described by Poulsen and co-workers [42].

#### Statistical analysis

Data analyses were performed using SPSS 21.0 (SPSS Inc., Chicago, IL, USA). In the statistical analyses, the primary outcome was the progression of AL≥3 mm in one or more sites at individual level, based on the collection of data performed at the baseline examination (in 2009) and at the follow-up examination (in 2011). The estimated risk associated with the progression of AL≥3 mm during a two-year follow-up period according to leukotoxicity determined by the three methods (cell lysis, cell death and haemolysis) was evaluated by calculation of odds ratios (OR). A value of *p*<0.05 was considered statistically significant.
